# Gonococcal Septic Arthritis of the Wrist in an Elderly Patient: A Case Report

**DOI:** 10.7759/cureus.117878

**Published:** 2026-10-13

**Authors:** Ruben Dias, Renato Rodrigues, Marta Silva, Pedro Balau, António Miranda

**Affiliations:** 1 Orthopedics and Traumatology, Unidade Local de Saúde de Entre Douro e Vouga, Santa Maria da Feira, PRT; 2 Orthopedic Surgery, Unidade Local de Saúde de Entre Douro e Vouga, Santa Maria da Feira, PRT; 3 Orthopedics, Unidade Local de Saúde de Entre Douro e Vouga, Santa Maria da Feira, PRT

**Keywords:** elderly, gonococcal arthritis, neisseria gonorrhoeae, septic arthritis, wrist

## Abstract

Gonococcal arthritis classically affects young, sexually active adults, and this profile shapes clinical suspicion. We report a 75-year-old man with hypertension and dyslipidaemia who presented with acute purulent monoarthritis of the right wrist. Synovial fluid was purulent (170,501 leukocytes/µL, 89.7% polymorphonuclear cells, crystals negative); the Gram stain showed Gram-negative diplococci, and culture of the joint fluid grew *Neisseria gonorrhoeae*, as did other samples taken at surgery. The isolate was reported as "sensitive" (drugs tested and minimum inhibitory concentration (MIC) values not available); no blood culture results are available. No skin lesions or tenosynovitis were documented, and a urine multiplex polymerase chain reaction (PCR) collected on day 7, after antibiotics had started, was negative, so dissemination was not established. He underwent arthrotomy and lavage on day 1 and received ceftriaxone 2 g daily until day 14, a single dose of azithromycin 500 mg, and, guided by susceptibility, oral ciprofloxacin for seven days. At three months, he was asymptomatic (Visual Analogue Scale (VAS) 0/10 and Disabilities of the Arm, Shoulder and Hand (DASH) 25/100). At one year, he had no complaints, full mobility, and no recurrence. Imaging showed advanced carpal osteoarthritis, so structural changes could not be attributed to infection. This case suggests that gonococcal septic arthritis should be considered in older patients with acute purulent monoarthritis; as a single case, it does not establish a treatment approach.

## Introduction

Septic arthritis is an orthopaedic emergency requiring prompt diagnosis and treatment to prevent irreversible joint damage. While *Staphylococcus aureus* and *Streptococcus pneumoniae* remain the most common causative organisms [1], *Neisseria gonorrhoeae* is an important but frequently overlooked cause, particularly outside its classic epidemiological profile of young, sexually active adults [2].

Gonococcal infection of a joint results from haematogenous spread from a mucosal site (disseminated gonococcal infection, DGI) and typically presents in one of two patterns: a triad of migratory polyarthralgia, dermatitis and tenosynovitis, or purulent monoarthritis, most often affecting the knee [3]. The absence of current genitourinary symptoms therefore does not exclude gonococcal arthritis, and a history of such symptoms may be overlooked when the joint is the presenting problem. Because the disease is associated with sexual activity in younger populations, clinicians may not consider it in older patients. Gonococcal arthritis has been described in several presentations [4,5].

Diagnosis is further complicated by the limited sensitivity of synovial fluid cultures for *N. gonorrhoeae*, often below 50%, owing to the organism's fragility [2]. Appropriate culture technique and, where available, nucleic acid amplification testing (NAAT) are therefore important, although NAAT is not universally validated for synovial samples [6]. Wrist involvement, although less common than knee involvement, has been described [7]. Reports of gonococcal wrist arthritis in older adults are limited. We describe such a patient to illustrate the diagnostic and management questions it raises, including interpretation of imaging in a degenerative joint and the limits of the work-up, and we distinguish gonococcal septic arthritis from confirmed disseminated infection.

## Case presentation

A 75-year-old man presented to the Emergency Department on day 1 (D1) with two to three days of severe pain in the right wrist (8/10 on the visual analogue scale, VAS [8]), progressive swelling, and marked functional limitation. His history included hypertension and dyslipidaemia, treated with perindopril and pravastatin, and he had no known drug allergies. He denied fever, trauma, insect bites, previous joint disease, and gout. No urethral discharge, dysuria, or sexual history was recorded at presentation; after surgery, he reported an episode of urethral discharge about one week before presentation.

Examination showed marked swelling and erythema of the right wrist (Figure 1), pain on active and passive movement, tenderness, no skin breakdown, preserved flexor and extensor tendon function, finger strength 4/5 on the Medical Research Council (MRC) scale [9], and no neurovascular deficit. His Disabilities of the Arm, Shoulder and Hand (DASH) score [10] was 63.3/100. No skin lesions, tenosynovitis, or other joint involvement were documented.

**Figure 1:**
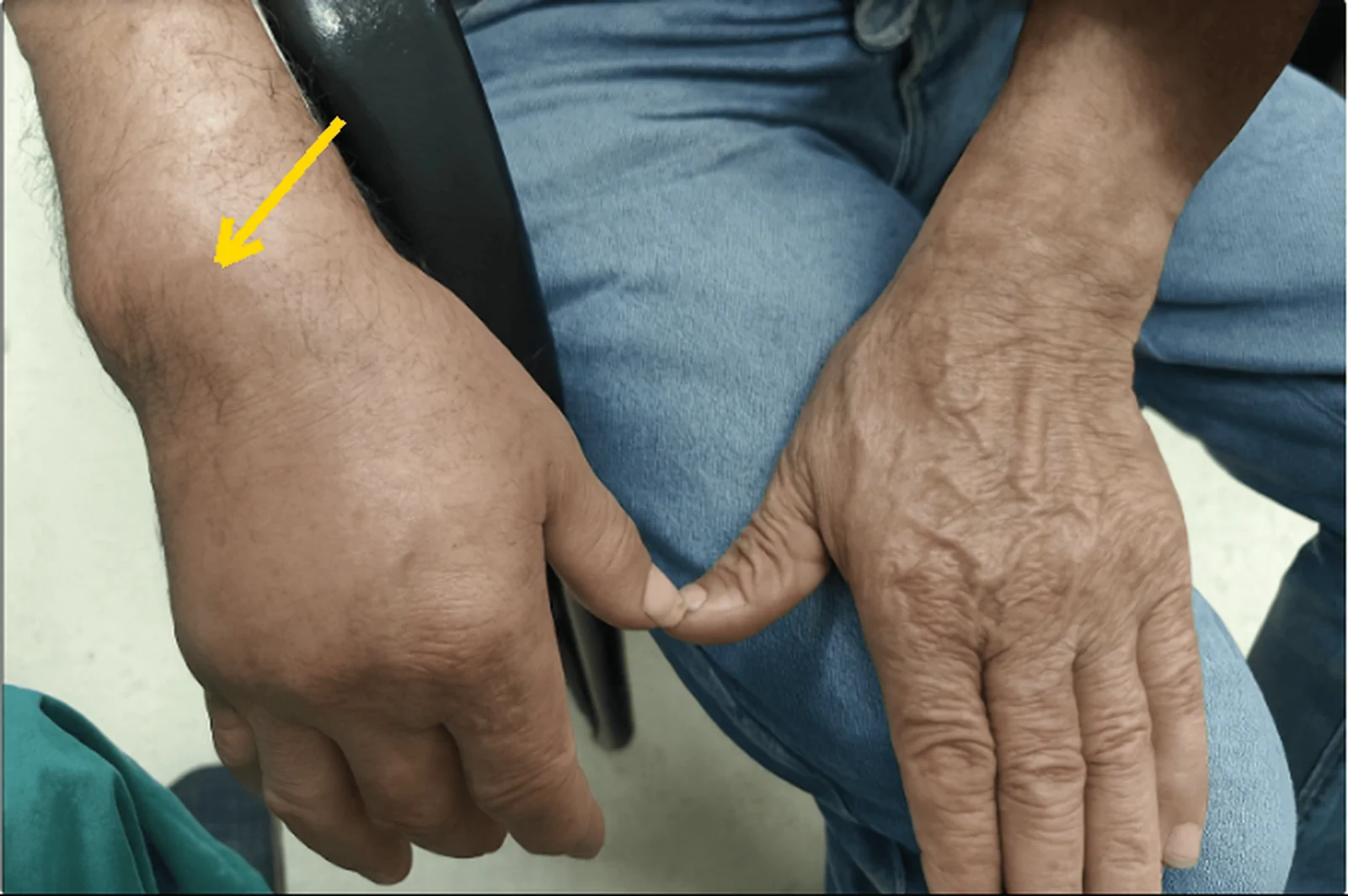
Right wrist at presentation (D1): swelling and erythema (arrow) compared with the contralateral wrist.

Laboratory tests on D1 (Table 1) showed leukocytosis with neutrophilia and a C-reactive protein (CRP) of 335 mg/L. Radiographs showed periarticular soft-tissue swelling and advanced degenerative changes of the radiocarpal and intercarpal joints, without fracture or focal bone lesion (Figure 2). Arthrocentesis on D1 yielded about 4 cc of purulent, turbid, pale-yellow fluid (170,501 leukocytes/µL, 89.7% polymorphonuclear cells, glucose 7 (unit not stated in the report), pH 7.20, total protein 5.2 g/dL), with no crystals under polarised light. The Gram stain showed polymorphonuclear leukocytes and Gram-negative diplococci (timing of the stain relative to surgery not documented).

**Table 1:** Laboratory and synovial fluid parameters, with reference ranges. CRP: C-reactive protein; D: day from admission (D1)

Parameter	Patient value	Reference range
Serum leukocytes (D1)	13.7 × 10⁹/L	4.0-11.0 × 10⁹/L
Serum neutrophils (D1)	84.8%	40-70%
Serum glucose (D1)	159 mg/dL	83-110 mg/dL
CRP (D1)	335 mg/L	<5.0 mg/L
CRP (D8)	26 mg/L	<5.0 mg/L
CRP (D25)	8.2 mg/L	<5.0 mg/L
CRP (D42)	5.4 mg/L	<5.0 mg/L
Synovial leukocytes (D1)	170,501/µL	<200/µL
Synovial polymorphonuclear cells (D1)	89.70%	<25%
Synovial erythrocytes (D1)	23,500/µL	-
Synovial glucose (D1)	7 (unit not stated)	Close to serum glucose
Synovial pH (D1)	7.20	7.3-7.4
Synovial total protein (D1)	5.2 g/dL	1.0-3.0 g/dL
Synovial crystals (D1)	Negative	Negative

**Figure 2:**
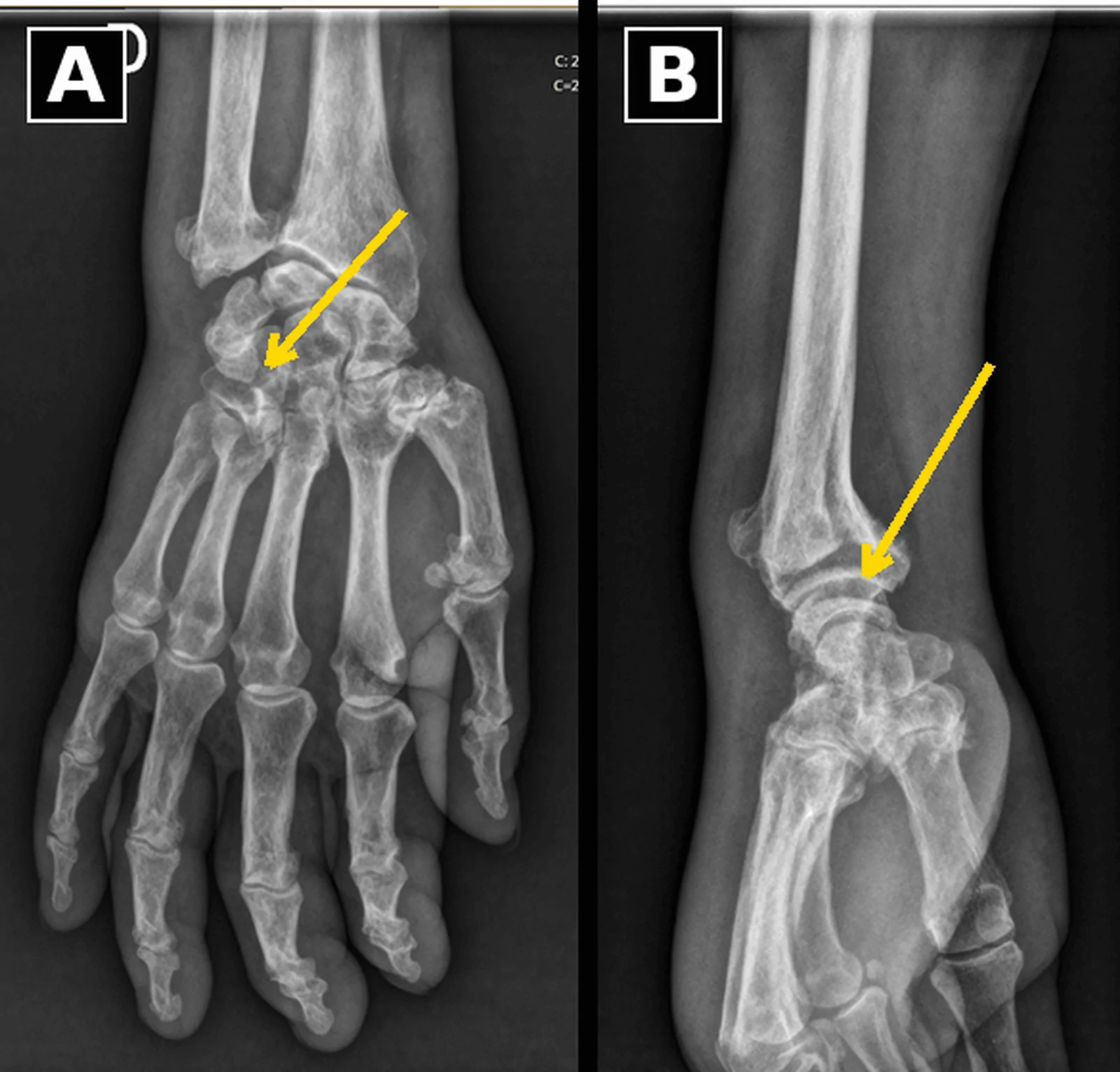
Radiographs of the right wrist on D1. (A) Anteroposterior and (B) lateral views, showing periarticular soft-tissue swelling and advanced degenerative changes of the radioscaphoid and radiolunate joints (arrows), without fracture. These changes pre-date or are independent of infection and cannot be attributed to it.

The same day, after multidisciplinary discussion and informed consent, he underwent dorsal arthrotomy between the third and fourth extensor compartments. Haemopurulent fluid was drained, synovial tissue was sampled, and the joint was irrigated; the wrist was immobilised in a volar splint. Four samples were sent for microbiology. Aerobic culture of the joint fluid grew *N. gonorrhoeae*, and the laboratory reported the same strain in the other samples. The identification method and the date the culture was reported are not available. HIV and syphilis serology were negative; susceptibility was recorded as "sensitive" without the report; no blood culture results are available.

Intravenous amoxicillin-clavulanate 2.2 g was started on D1 per the department's protocol and, once *N. gonorrhoeae* was isolated, changed to intravenous ceftriaxone 2 g every 24 hours starting on the second postoperative day (D2 or D3). A single oral dose of azithromycin 500 mg was added after discussion with the internal medicine team. A urine multiplex polymerase chain reaction (PCR) collected on D7 did not detect *N. gonorrhoeae*, *Chlamydia trachomatis*, *Treponema pallidum*, or other targets. Anti-*N. gonorrhoeae* antibodies (complement fixation) were negative on D42.

He improved progressively. CRP was 26 mg/L on D8, 8.2 mg/L on D25, and 5.4 mg/L on D42 at the outpatient visit (reference <5.0 mg/L), that is, from 335 mg/L at presentation to close to normal, with normal leukocyte counts on D25 and D42. Intravenous ceftriaxone was given until D14 (13 days), and on D14 the dressing was dry, with painless range of motion and no significant effusion; treatment was changed to oral ciprofloxacin 500 mg twice daily for seven days (20 days in total), as the isolate was reported as "sensitive" (drugs tested and minimum inhibitory concentration (MIC) values not available). Computed tomography (CT) on D35 showed high-grade chondral erosions of the radioscaphoid and radiolunate compartments with subchondral cysts in several carpal bones, scaphotrapeziotrapezoid erosions, advanced trapeziometacarpal osteoarthritis, a small effusion with diffuse periarticular oedema, and distal radioulnar osteoarthritis with positive ulnar variance, without carpal instability (Figure 3). Because advanced degenerative disease was already present, the contribution of infection to these findings cannot be determined. Magnetic resonance imaging (MRI) on D43 was severely limited by artefact and was not evaluable at the wrist; the report described possible, inconclusive signs of septic arthritis (Figure 4). The sequence of events is summarised in Table 2.

**Figure 3:**
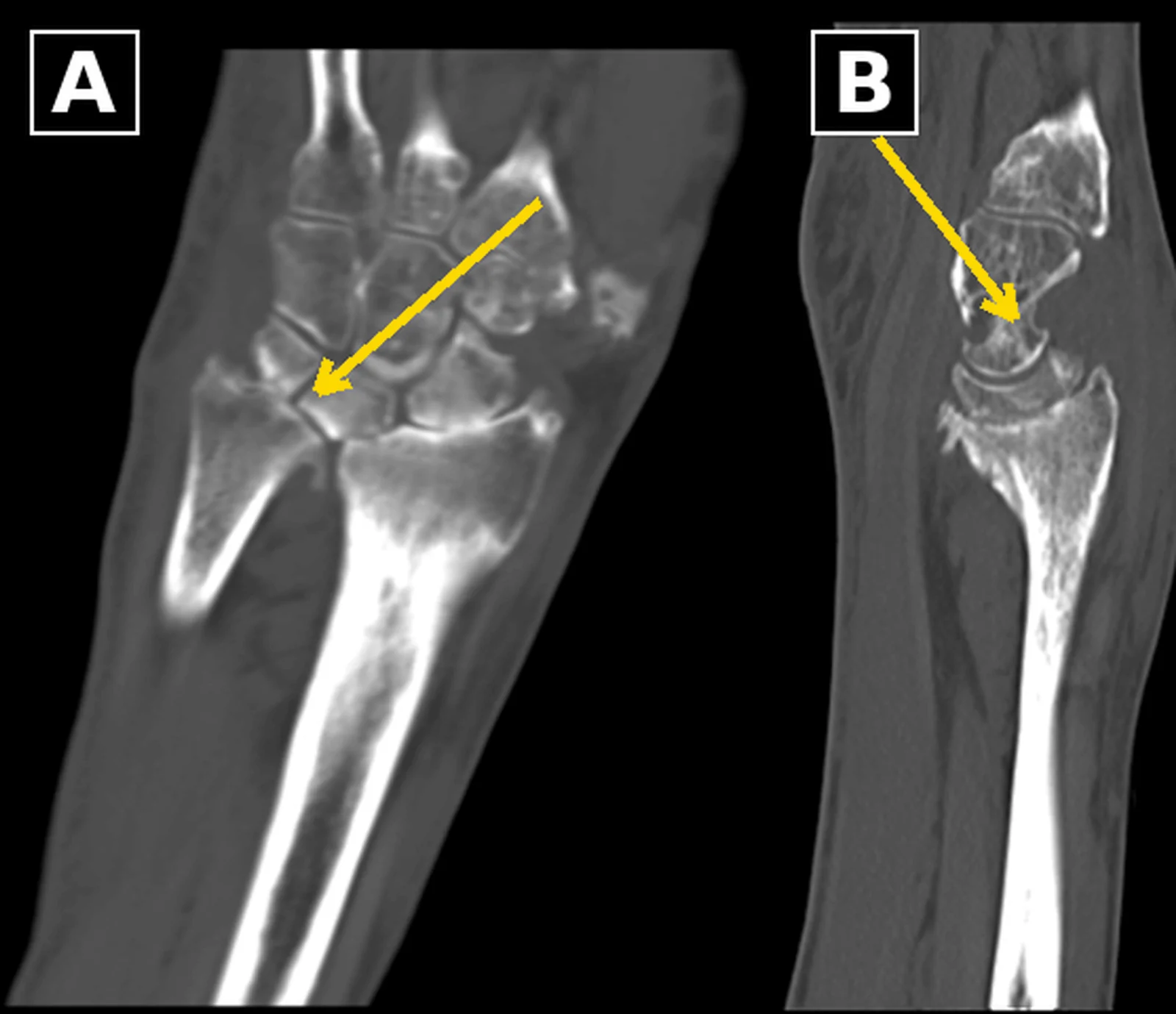
Computed tomography of the right wrist on D35. (A) Coronal and (B) sagittal reconstructions, showing chondral erosions and subchondral cysts at the radiocarpal joint (arrows) on a background of advanced degenerative disease; the contribution of infection cannot be determined.

**Figure 4:**
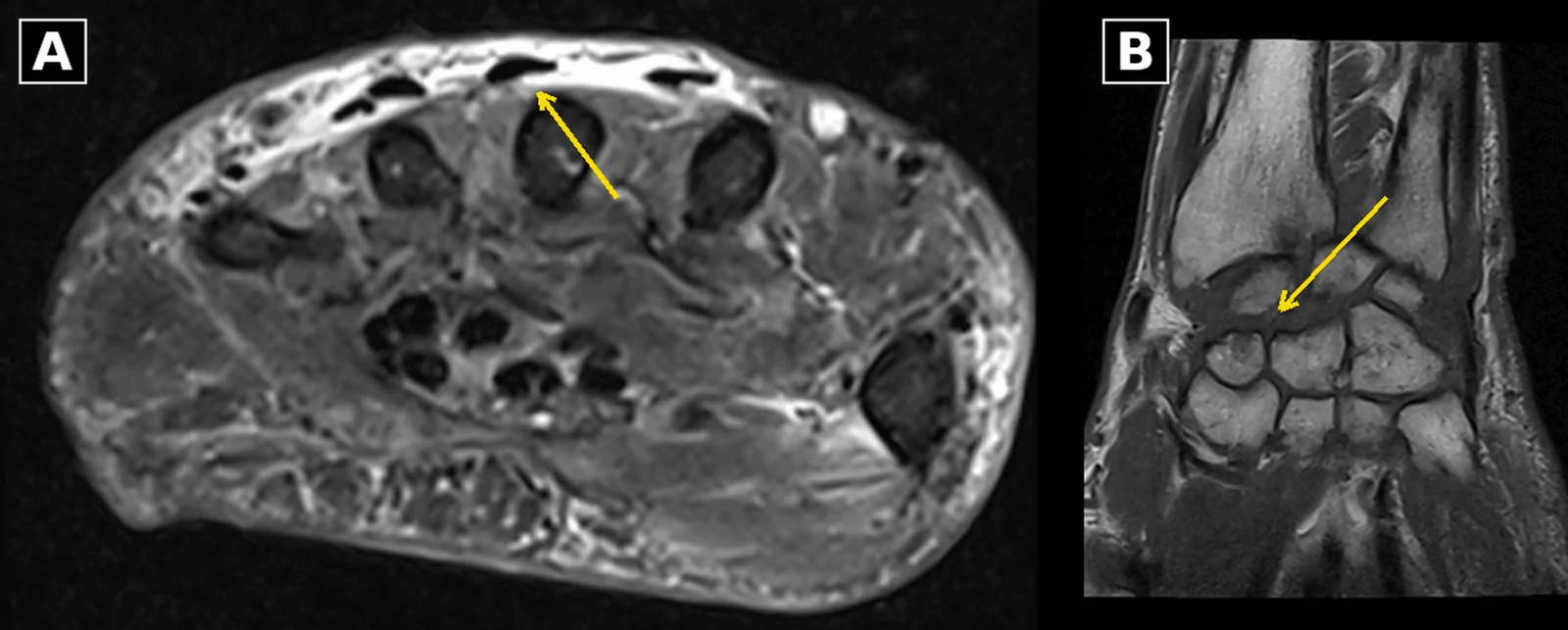
Magnetic resonance imaging of the right wrist on D43. (A) Axial and (B) coronal. The wrist is severely affected by artefact, and the study is inconclusive.

**Table 2:** Timeline of events. D1 is the day of admission. CRP: C-reactive protein; CT: computed tomography; DASH: disabilities of the arm, shoulder, and hand; IV: intravenous; MRI: magnetic resonance imaging; PCR: polymerase chain reaction; VAS: visual analogue scale

Day	Event
About D-7 (reported by the patient after surgery)	Urethral discharge (patient report; no genitourinary examination or testing before antibiotics)
D-3 or D-2 (2 to 3 days of pain)	Onset of right wrist pain and swelling
D1	Presentation, blood tests, arthrocentesis (purulent fluid, Gram-negative diplococci), arthrotomy and lavage, four samples to microbiology, IV amoxicillin-clavulanate
D2 (or D3)	*N. gonorrhoeae *isolated; IV ceftriaxone 2 g every 24 hours; azithromycin 500 mg, single dose
D7	Urine multiplex PCR: negative
D8	CRP 26 mg/L; ceftriaxone continued
D14	Dressing dry, painless range of motion; ceftriaxone completed (13 days); oral ciprofloxacin 500 mg twice daily for 7 days started (isolate susceptible)
D25	CRP 8.2 mg/L; leukocytes 4.4 × 10⁹/L
D35	CT: erosions and cysts on advanced carpal osteoarthritis
D42	CRP 5.4 mg/L; anti-*N. gonorrhoeae* antibodies negative
D43	MRI: severe artefact, not evaluable
3 months	No symptoms, VAS 0/10, no stiffness or instability, DASH 25/100
1 year	No complaints, full wrist mobility, no recurrence; no DASH, imaging or laboratory data

At three months, he had no symptoms, VAS 0/10, no stiffness or instability, and DASH 25/100 (Figure 5). At the one-year review, he remained asymptomatic, with full range of motion and no recurrence (no DASH, imaging, or laboratory data from that visit). The visits took place three months and one year after surgery, and the patient completed the DASH at the clinic visit. Inflammatory markers were not measured at the three-month or one-year visits; the last value, at the D42 outpatient visit, was CRP 5.4 mg/L (reference below 5.0 mg/L), close to normal. 

**Figure 5:**
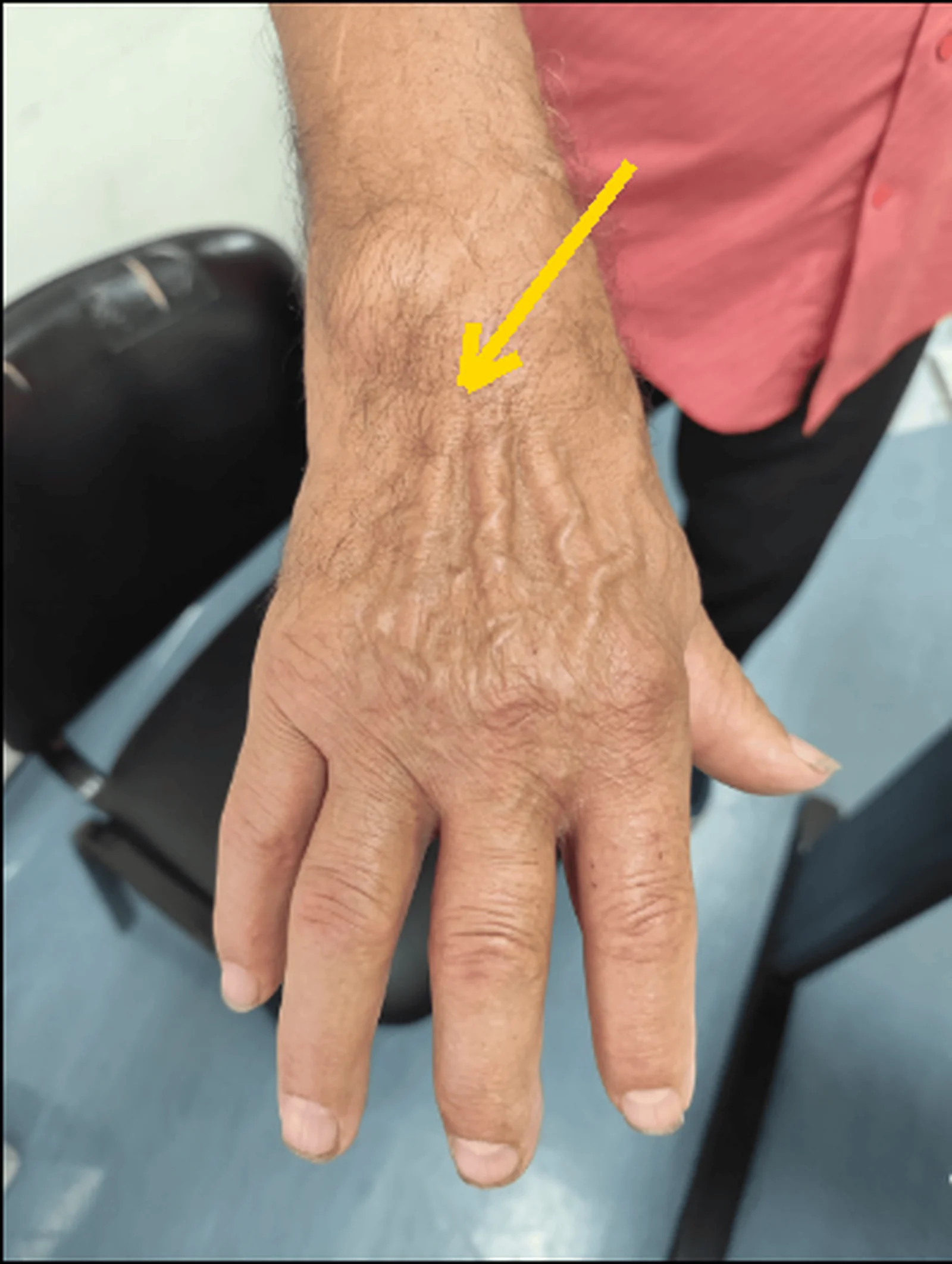
Right wrist at 3 months, with no swelling or erythema at the previously affected site (arrow).

## Discussion

Three features make this case unusual: the patient's age, isolated involvement of the wrist, and a joint with advanced pre-existing degenerative disease. Gonococcal arthritis is typically described in young, sexually active adults and most often affects the knee [3]. A published report describes gonococcal arthritis of both wrists and hands in a man in his seventies, in whom diagnosis was delayed by non-diagnostic aspirations [11], and wrist involvement has been reported in other patients [7,12]. In our patient, the diagnosis was reached at the first arthrocentesis because the Gram stain and culture of joint fluid were positive. The case adds to a limited literature on older adults but cannot show how often this occurs.

Gonococcal arthritis is attributed to haematogenous spread from a mucosal site, and purulent monoarthritis without skin lesions is a recognised presentation of disseminated infection [3]. In our patient, however, dissemination was not established: no blood culture results are available, no skin lesions or tenosynovitis were documented, and the urine PCR was collected after antibiotics had started. We therefore describe the case as gonococcal septic arthritis. A genital culture or NAAT before treatment would have helped to identify a mucosal source.

The urethral discharge reported by the patient after surgery suggested a sexually transmitted cause, but it was only recorded retrospectively and did not identify the organism; the diagnosis rested on the joint fluid Gram stain and culture. Crystal arthritis was excluded by negative polarised-light microscopy. Non-gonococcal bacterial arthritis, the commonest cause [1], was addressed by culture, which grew *N. gonorrhoeae*. Culture sensitivity for *N. gonorrhoeae* is limited, often below 50% [2], and NAAT may help when cultures are negative, although it is not universally validated for synovial samples [6].

Advanced degenerative disease of the radiocarpal, intercarpal, trapeziometacarpal and distal radioulnar joints was visible on admission radiographs and on CT. It may have predisposed to infection, and it prevents attributing the erosions and cysts on CT to infection. The CT was obtained five weeks after surgery and did not change management, and the MRI was not evaluable. Imaging therefore did not establish infection in this patient; the diagnosis rested on microbiology.

The CDC guideline recommends, for DGI, ceftriaxone 1 g every 24 hours, with a switch to an oral agent guided by susceptibility testing 24-48 hours after substantial clinical improvement and at least seven days in total, and doxycycline for seven days if chlamydia has not been excluded [6]. Our patient's management differed. Ceftriaxone was given at 2 g daily for 13 days, and treatment lasted 20 days in total. Oral ciprofloxacin was used for step-down after the isolate was reported susceptible (drugs tested and MIC values not available), which is consistent with the guideline's advice to guide oral therapy by susceptibility; the guideline names no oral agent, and fluoroquinolones have not been recommended for empirical gonorrhoea treatment in the United States since 2007 because of resistance [6,13], so this approach is appropriate only with documented susceptibility. A single 500 mg dose of azithromycin was given (the reason is not recorded), which is neither the preferred chlamydia regimen (doxycycline for seven days; azithromycin 1 g is an alternative) nor part of current gonorrhoea treatment [6]. The urine PCR did not detect *C. trachomatis*, although collected after antibiotics. Surgical drainage was performed on D1; the guideline does not address drainage, so this reflects local practice for purulent septic arthritis. The patient recovered, but these decisions describe one patient and are not recommendations.

Regarding residual disability, the DASH score fell from 63.3 to 25 at three months. Residual limitation may reflect carpal osteoarthritis, a post-infectious cause, or age, and these factors cannot be separated. At one year, there was no recurrence, and mobility was full; the follow-up has the limitation that no imaging, laboratory, or DASH data are available after three months.

This report has limitations. It describes one patient, so it cannot establish frequency, generalisability, or a treatment approach. No blood culture results are available, and although HIV and syphilis serology were negative, immune status and dissemination could not be fully assessed, and "immunocompetent" is not claimed. No genitourinary specimen was tested before antibiotics. The wrist already had advanced osteoarthritis; the MRI was not evaluable, and the urine PCR was collected after antibiotics. Follow-up data are limited to the clinical review at three months (VAS and DASH) and at one year (clinical examination only).

## Conclusions

This case suggests that gonococcal septic arthritis should be considered in older patients with acute purulent monoarthritis, even without a typical risk profile. It highlights the value of a genitourinary history, of obtaining joint fluid and other specimens for Gram stain, culture, and susceptibility testing before antibiotics where possible, and of choosing antimicrobials according to clinical and microbiological findings. As a single case with pre-existing joint disease and a limited work-up, it does not establish a treatment approach, and dissemination was not confirmed.
